# Retraction: Luciferase-transfected colon adenocarcinoma cell line (DLD-1) for use in Orthotopic Xenotransplantation studies

**DOI:** 10.1186/1752-153X-6-121

**Published:** 2012-10-22

**Authors:** Muhammad Rashid Siddique, Steve Shynder, Muhammad Aqeel Ashraf, Ismail Yusoff, Abdul Wajid

**Affiliations:** 1 Guy Hilton Research Centre, Keele University, Staffordshire, UK; 2 Department of Chemistry, University of Malaya, 50603, Kuala Lumpur, Malaysia; 3 Department of Geology, University of Malaya, 50603, Kuala Lumpur, Malaysia; 4 Deprtment of Chemistry, The Islamia University of Bahawlapur, Bahawlapur, 63100, Pakistan

## 

Retraction

This article is retracted.

The data in this article [1] was used without permission by the lead author Muhammad Rashid Siddique.

The lead author accepts full responsibility and would like to apologize to the owners of the data (Dr Steve Shnyder, University of Bradford, UK), as well as the co-authors of this article, the Editors and readers.
